# Commissioning and performance characteristics of a pre-clinical image-guided radiotherapy system

**DOI:** 10.1007/s13246-019-00755-4

**Published:** 2019-04-15

**Authors:** Theresa V. Feddersen, Pejman Rowshanfarzad, Tamara N. Abel, Martin A. Ebert

**Affiliations:** 1 Department of Physics, University of Western Australia, Crawley, WA Australia; 2 Telethon Kids Institute, University of Western Australia, Subiaco, WA Australia; 3 Department of Radiation Oncology, Sir Charles Gairdner Hospital, Nedlands, WA Australia; 4 Department of Radiotherapy, Erasmus University Medical Center, Rotterdam, The Netherlands; 5 Department of Radiology & Nuclear Medicine, Erasmus University Medical Center, Rotterdam, The Netherlands

**Keywords:** Small animal radiotherapy, Pre-clinical, Commissioning, Dose delivery, Image guidance

## Abstract

Characteristics of a small-animal radiotherapy device, the X-RAD SmART, are described following commissioning of the device for pre-clinical radiotherapy research. Performance characteristics were assessed using published standards and compared with previous results published for similar systems. Operational radiation safety was established. Device X-ray beam quality and output dose-rate were found to be consistent with those reported for similar devices. Output steadily declined over 18 months though remained within tolerance levels. There is considerable variation in output factor across the international installations for the smallest field size (varying by more than 30% for 2.5 mm diameter fields). Measured depth dose and profile data was mostly consistent with that published, with some differences in penumbrae and generally reduced flatness. Target localisation is achieved with an imaging panel and with automatic corrections for panel flex and device mechanical instability, targeting within 0.2 mm is achievable. The small-animal image-guided radiotherapy platform has been implemented and assessed and found to perform as specified. The combination of kV energy and high spatial precision make it suitable for replicating clinical dose distributions at the small-animal scale, though dosimetric uncertainties for the narrowest fields need to be acknowledged.

## Introduction

It is problematic to evaluate alterations to radiotherapy treatments, including in combination with new drugs, on human populations. Problems include unknown or unexpected interactions and adverse effects, the lengthy and complex clinical trial process, ethics issues, the large number of subjects required to achieve statistical significance for an intervention presenting clinical equipoise, and the difficulties of undertaking detailed post-treatment anatomical studies. Pre-clinical studies can be undertaken on small animals to mitigate these difficulties, with devices for delivering high-precision radiotherapy treatments on small animals now commercially available. These devices attempt to mimic the dose deliveries achievable with clinical units and are considered a major step in radiobiology research [1].

Over the last two decades, significant advances in radiotherapy technology have enabled increasingly sophisticated methods for treatment planning, delivery and imaging to be implemented into routine radiation oncology practice [2–4]. Several groups have developed small animal (3D) image-guided radiotherapy devices, which similarly allow precise irradiation of structures in small animals [2, 5, 6], thus bridging the gap between preclinical and clinical radiotherapy technology. However, the commissioning of kV small field devices is lacking published data [4].

Verhaegen et al. highlighted the efforts of five research groups and compared the characteristics of their developed systems [6]. The photon energy investigated ranged between 5 and 380 keV, with field sizes ranging from 0.5 mm to 20 mm diameter. Most institutions could use fixed fields as well as arcs for the irradiation. Targeting accuracy varied from 0.065 mm to 0.2 mm. Recommendations were made for the ideal requirements for an image-guided small animal irradiator which included a targeting accuracy of ± 0.1–0.3 mm depending on the type (size) of animal treated.

The aim of this study was to report on the commissioning and performance characteristics of the first dedicated pre-clinical image-guided radiotherapy (IGRT) system in Australasia. This study followed acquisition of a commercial small-animal IGRT system, the X-RAD SmART (Precision X-ray, North Bramford CT), at the Telethon Kids Institute in Perth, Western Australia. Newton et al. commissioned the same device incorporating point, 2D and 3D measurements and concluded independent 2D and 3D measurements to be valuable to ensure accurate and comprehensive commissioning [7]. Lindsay et al. [8] previously compared the dosimetric and geometric properties of X-RAD devices installed at three institutions. The commissioning procedure defined by Lindsay et al. was replicated in the present study and, where possible, measurement results were compared with those from Lindsay et al. and other published data. This provides some perspective on the operating characteristics of these devices, the variability of those characteristics between devices and the likely uncertainties in reported experimental parameters.

## Materials and methods

### Pre-clinical IGRT system

**Fig. 1:**
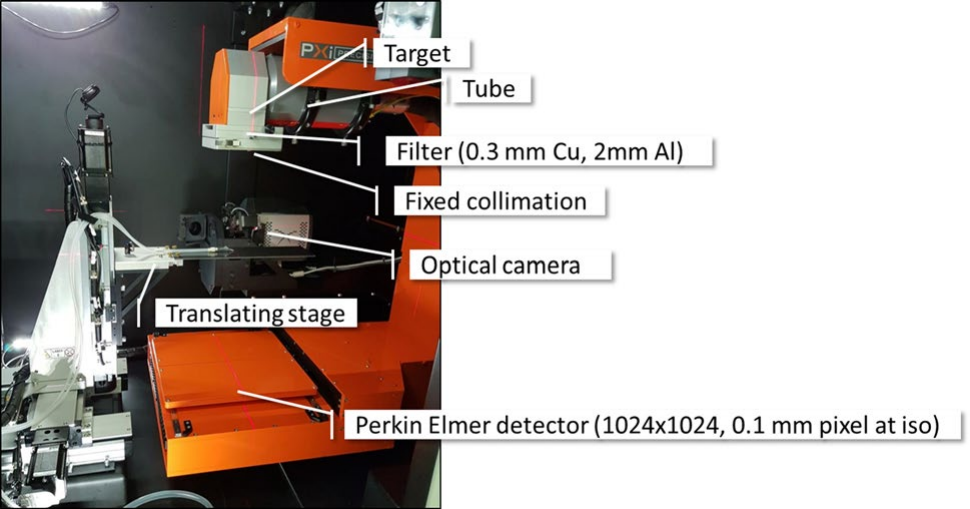
X-RAD device components

The X-RAD has a dual focal-spot X-ray tube mounted on a rotating gantry at a source-to-isocentre distance of 306.4 mm, as can be seen in Fig. 1. Gantry angles are defined with 0° corresponding to the source at the top of rotation through to + 360° over clockwise rotation when facing the gantry axis. X-ray energies up to 225 kVp can be generated with either 0.3 mm Cu or 2 mm Al filters (typically used for therapy and imaging, respectively), with manually attached collimators defining 2.5, 5, 10 and 25 mm diameter circular fields and 10 × 10 mm^2^ and 40 × 40 mm^2^ square fields (dimensions at isocentre). A Perkin Elmer amorphous silicon imaging panel is mounted on the counter side of the gantry and is used for image-based localization using cone-beam computed tomography (CBCT) or planar imaging as well as for verifying beam targeting (resolution ~ 0.1 mm at isocentre). A remotely-controlled platform allows for positioning of the animal relative to the beam. Beam delivery can be performed by either static beams or arc rotation. The system sits in a fully self-shielded cabinet. The system installed at the Telethon Kids Institute has an optical camera installed orthogonal to the X-ray beamline for bioluminescence studies. The X-RAD SmART is controlled by Pilot software version 1.12.

Treatment planning is achieved with SmART-Plan version 1.3.12 installed on a Dell Precision Tower 5810 running on Ubuntu 12.04.1 LTS 64 bit. This system allows manual design of beams with a Monte Carlo dose calculation performed on cone-beam CT images acquired using the Perkin Elmer panel. The Monte Carlo algorithm utilises pre-calculated phase space files, for each collimator, provided by the manufacturer. Absolute calibration of the dose calculation is made by matching calculated dose output to the device absolute output measurement. This can require applying some output corrections, especially for the smallest collimators, to account for the effect of slight misalignment of the X-ray source and collimator. For the Telethon Kids Institute unit, a correction of 0.93 was applied for just the 2.5 mm diameter collimator.

### Radiation survey

**Fig. 2:**
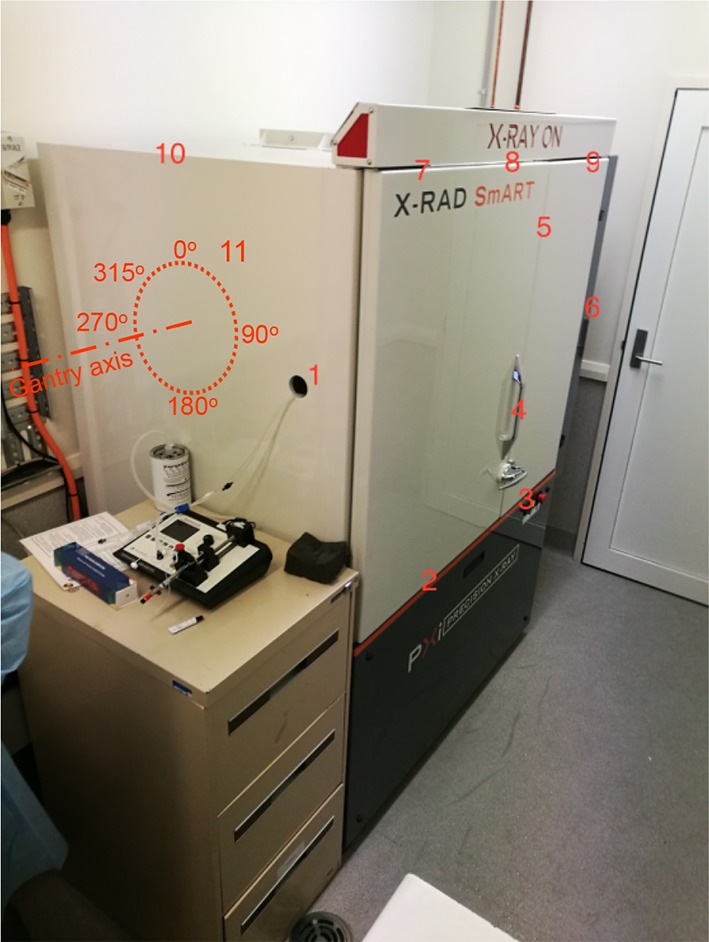
The location of the 11 survey points investigated during radiation safety survey. Point 11 remained aligned with the X-ray tube cathode–anode axis (gantry angle indicated)

A radiation survey was undertaken around the cabinet to test for leakage. The device was operated with the typical therapy settings (225 kV, 0.2 mm Cu filter at 13 mA) with the gantry at 0°, 180°, 225°, 270°, 315°. A calibrated, pressurised *µ*R ion chamber survey meter (Fluke 451, s/n 451P-DE-SI-RYR, Fluke Biomedical, Cleveland OH) was used for obtaining the dose rates at 11 points external to the device cabinet as shown in Fig. 2. The highest rate was found on the side of the cabinet in alignment with the X-ray tube cathode–anode axis, and as such point 11 rotates with the gantry.

### Absolute dosimetry

#### Half value layer (HVL)

HVL measurements were made with the gantry at 180° with an IBA FC65-G (3502) chamber (IBA Dosimetry GmbH, Schwarzenbuck, Germany), connected to a Sun Nuclear 1014000-1Z PC electrometer (Sun Nuclear, Melbourne FL), positioned with its long axis at the gantry rotation axis. HVL was measured for 225 kV, 13 mA, large focal spot and using the Cu (treatment) filter by adding sheets of Cu (99.9% purity) to the beam exit window. The first and second HVLs were measured.

#### Output dose-rate

Primary measurement of the output dose-rate for the device was according to the protocol defined by American Association of Physicists in Medicine (AAPM) Task Group 61 (TG-61) protocol for 40–300 kV X-ray dosimetry [9]. Measurement was made with a PTW TN30013 Farmer-type ion chamber in air.

Some modifications had to be made to the AAPM TG-61 protocol, due to the difference in dimensions and accessibility compared to a clinical machine. The square 40 × 40 mm^2^ field was used instead of the recommended 10 × 10 cm^2^ field. Calibration was also performed at a source to detector distance of 306.4 mm rather than the prescribed 100 cm due to the confines of the cabinet. Chamber positioning was achieved using the CBCT imaging facility.

Following calibration, a constancy measurement was made in-air using the FC65-G (3502) chamber and PC electrometer. This chamber was used for all subsequent measurements. Output constancy was then assessed at subsequent routine QA sessions occurring approximately bi-monthly.

#### Linearity

To investigate the output dose-rate linearity with current and time, in-air measurements were taken at the following settings: 225 kVp, Cu filter, at 1, 2, 5, 10, 30 and 60 s and 13, 10, 8, 6, 4, 2, 1, 0.5, 0.2, 0.1 mA. The same ion chamber, electrometer combination as described above was used, with the chamber positioned with its long axis on isocentre. The data was recorded and two curves were fitted: charge *vs* beam-on time and charge *vs* current. From the charge *vs* beam-on time graph the timer error (also known as the end-effect) was assessed. This was achieved by extrapolating the linear fit of the measured points and observing at which value it crossed the y-axis.

### Relative dosimetry

#### Film dosimetry

For the characterisation and verification of dose distributions, radiochromic film (RCF) was used because of its 2D high-resolution dosimetric capabilities. The Gafrochromic EBT3 (Ashland Advanced Materials, Bridgewater NJ) film was used. In order to define a quantitative relationship between the optical density of the film and the dose delivered, a calibration curve was established. Pieces of the same badge of film used for the experiment later were placed at isocentre perpendicular to the beam and irradiated for various beam-on times. From this, after adjusting for the measured output of the day, the incident dose (in Gy) was found.

In order to find a quantitative relationship between the optical density and the dose delivered, a calibration curve was established. The settings of the X-RAD for the calibration were 225 kV, 13 mA, large spot size with the 40 × 40 mm^2^ collimator and the therapy filter (Cu). Pieces of film of approximately 4.5 × 3 cm^2^ were placed at the isocentre perpendicular to the beam on the surface of a 5 cm thickness of solid water slabs (RW3, IBA Dosimetry, Schwarzenruck, Germany). The tendency for RW3 to underestimate percentage dose at depth [1] is acknowledged. Specific details of solid water used were not provided in the report by Lindsay et al. [8]. Irradiation at gantry 0° was performed for various beam-on times and associated calculated doses. For each dose, three films were irradiated to assess uncertainty.

The films were scanned into Sun Nuclear SNC Patient software (Sun Nuclear, Melbourne FL) 24 h after exposure using an Epson 10000 × l scanner with transparency unit. SNC Patient requires a scan setting of 48 bit and 75 dpi (being the clinical standard set up), with all colour corrections turned off. The same batch of RCF was used for all measurements and was handled according to the recommendations outlined in the AAPM TG-55 report [10]. The resulting images were then analysed in MATLAB for red, blue and green channels and a curve was fitted to the red channel response. The resulting relationship between intensity of the film and dose was used to process all other measurements.

The relative dosimetry measurements were made using EBT3 Gafchromic film in a solid water phantom, unless stated otherwise. The phantom consisted of solid water slabs with 1 cm and 0.5 cm thickness, positioned on the detector plate. EBT3 film was cut into approximately 5 × 5 cm^2^ pieces and inserted in between the slabs starting at the surface, from at 5 mm depth and then every 1 cm until a maximum depth of 5.5 cm was reached. The set-up was such that the surface of the solid water was at the isocentre of the device. The phantom was then irradiated for 120 s for every collimator. Seven films were evaluated per irradiation and the process was repeated three times for each collimator. All measurements were performed with the film being perpendicular to the beam axis. The repeat measurements allow for the determination of measurement standard deviation, with uncertainties resulting from stochastic output changes, as well as inherent differences in the film, scanner and handling, even though care was taken to keep these to a minimum by following the standard practices when handling the RCF.

The irradiated films were subsequently scanned 24 h after exposure, and imported into MATLAB as TIFF images. In the processing and analysis of the images, adaptive filtering was performed to reduce the noise. This was done using the wiener2 function in MATLAB, which uses a pixel- wise adaptive Wiener method based on statistics estimated from a local neighbourhood of each pixel. Neighbourhoods of size [5] were used. Depending on the beam property investigated, either a region of interest was chosen from the films or the central values from which dose profiles could be extracted.

#### Percentage depth dose (PDD)

To calculate the PDD, the central ROI signal at 0 cm solid water depth was extracted from the corresponding films, averaged and taken as the reference. The ROI values of all other depths were obtained in the same manner and then compared to the reference to obtain the PDD (for each collimator).

#### Relative output factor (ROF)

The relative output factor was measured at a depth of 0.5 cm. The reference field for the relative output factor measurement was 40 × 40 mm^2^ field. The ROF’s were calculated from:

1 $$ROF_{col} = \frac{{D_{col} }}{{D_{ref} }}$$

where D_col_ and D_ref_ are the dose with a given collimator and the dose at the reference collimator respectively (both at solid water depth of 0.5 cm). To calculate the ROF the central region of interest values of the TIFF images were used as defined by Lindsay et al.

#### Dose profiles

The profiles were taken through the central values in the x and y direction (cross-plane and in-plane) of the field. Each profile is the average of five adjacent profiles to provide better statistics. From these profiles, symmetry, field size at full width half maximum (FWHM) and the penumbra were obtained for all available field sizes. The field size is defined by the region of 50% or more of the signal, normalised to the signal on central axis (the FWHM). Each penumbra was evaluated as the distance between the 80 and 20% of the signal.

Symmetry and flatness were both assessed within the central 80% of the field, a region bounded by 80% of the full field width. Symmetry of the profile is defined as the maximum value of the ratio of D_−x_ to D_+x_:

2 $${\text{Symmetry}} = { \hbox{max} }\left| {{\text{D}}_{{ + {\text{x}}}} - {\text{D}}_{{ - {\text{x}}}} } \right| *100\%$$

D_−x_ represents the signal at a distance x to one side of the central axis of the beam and D_+x_ is the signal at the corresponding point on the other side. Where, + x and − x are symmetric pixels starting closes to and then making their way further away from the central pixel.

For the flatness, the same field as for the symmetry is considered and the following is investigated:

3 $$Flatness = \frac{{D_{max} - D_{min} }}{{D_{max} + D_{min} }}*100\%$$

where D_max_ and D_min_ are the maximum and minimum dose respectively of the central 80% profile as defined above.

The offset of the source from isocentre can be obtained using the beam penumbra in the in-plane and cross-plane direction. By subtracting the left from the right penumbra in both planes and then plotting the resulting values on a 2D coordinate system, any systematic shift will be visible. This shift is coupled to any limits in tolerance in physical construction of the collimation system, and so is referred to here as an “effective offset”.

#### Inverse square law

The dose fall-off with distance should follow the inverse square law if the X-ray source represents a point. Due to additional source components, such as scatter from the cabinet walls, the inverse-square behaviour may be disrupted. To determine the extent of this effect, the ion chamber was fixed at isocentre and then irradiated three times at 225 kVp, 13 mA using the 40 × 40 mm^2^ collimator and an average was taken. This was repeated at + 5 cm, + 3 cm, − 3 cm and − 5 cm from isocentre by moving the couch vertically (y-direction) only. To calculate the effective SSD:

4 $$\left( {\frac{{D_{0} }}{{D_{\Delta } }}} \right)^{2} = 1 + \frac{\Delta }{{SSD_{eff} }}$$

where D_0_ is the dose at zero y offset of the couch, $D_{\Delta }$ is the dose at a certain offset in y of $\Delta$, and $SSD_{eff}$ is the effective SSD. $SSD_{eff}$ was found by obtaining linear fit to a plot of $D_{\Delta }$ against $\Delta$ and applying Eq. 4.

#### Simulation of solid water with planning system

In order to simulate dose delivery in the solid water slabs using the Monte Carlo software and compare planned versus delivered distributions, a digital solid water phantom was made (RW3—7.59% H, 90.41% C, 0.8% O, 1.2% Ti; 1.06 g cm^−3^) and imported into the treatment planning program for the X-RAD (SmART-Plan). Note that to ensure accurate simulation of scatter conditions, the simulated phantom had to replicate the physical dimensions of the physical phantom. Dose calculation voxels were 0.25 mm wide in each dimension normal to the beam axis and 1 mm along the depth axis. The same exposures that were used for measurements were simulated and the dose was exported into DICOM files. A Monte Carlo simulation dose calculation uncertainty (variance) of 1% to the highest dose voxel was specified. The DICOM files created were imported and analysed using MATLAB. The measurements for ROF, field size, penumbra, symmetry and flatness were obtained using the same methods as for the RCF data above. The results were obtained from slices at the same depths that were irradiated with the RCF.

The SmART-Plan system reports dose-to-medium and film was not included in the simulation. In comparison with relative dose values, an assumption is made that ratios of doses to the RW3 medium are equivalent to ratios of dose to film at the same points.

### Mechanical operation and performance

The mechanical tests were conducted using a BB (ball bearing) attached to a narrow Perspex rod. Positioning and movement of the ball bearing relative to the beam and imaging axes is determined automatically using manufacturer-supplied software, at the precision of individual image pixels (0.1 mm at isocentre).

#### Magnification

The Pilot software provides an automated process for assessing magnification factor, source to axis distance (SAD) and source to (panel) detector distance (SDD). This process utilises the BB attached to the remotely-controlled stage, imaged with the Perkin Elmer a-Si panel.

#### Panel flex

Assessment of flex of the imaging panel with gantry rotation is made by imaging the BB in multiple fixed positions during complete 360° gantry rotations. The resulting “projection” maps or “flexmaps” of the lateral (*u*) and longitudinal (*v*) flex of the panel were acquired for both the small and large focal spot sizes. The Pilot software stores measured flexmaps for correcting subsequent acquired images.

#### Isocentre stability

Changes in the device isocentre position and collimator alignment with gantry rotation were assessed using the “Winston-Lutz” calibration. A single BB was positioned at the machine isocentre and its position relative to the collimator-defined beam during a full 360° gantry rotation was measured. The Pilot software generated the resulting (u, v) displacements required to ensure the BB remains at the centre of collimation. These displacements, and the flexmap calibration, were applied to a repeat Winston-Lutz calibration to assess the overall accuracy of target tracking during gantry rotation.

## Results

### Radiation Survey

The background radiation was measured to be 0.05 μSv/h. The surveyed dose rates are presented in Table 1. None of the values are significantly larger than background, except for adjacent to the cabinet in alignment with the X-ray tube cathode–anode axis.

**Table 1:** Dose rate (μSv/h)—background dose rate 0.05 μSv/h at the surveyed points minus background radiation, for five different gantry angles (G)

Point	G = 0°	G = 315°	G = 270°	G = 225°	G = 180°
1	0.13	0.10	0.14	0.08	0.10
2	0.10	0.08	0.13	0.05	0.10
3	0.03	0.10	0.05	0.07	0.08
4	0.08	0.06	0.08	0.06	0.08
5	0.00	0.05	0.01	0.06	0.02
6	0.09	0.05	0.06	0.03	0.02
7	0.06	0.04	0.09	0.09	0.11
8	0.08	0.15	0.12	0.06	0.13
9	0.05	0.09	0.06	0.09	0.06
10	0.21	0.15	0.12	0.07	0.10
11	0.81	0.86	0.83	0.70	0.25

G = 0° means the X-ray source is irradiating vertically downwards. G = 270° means the X-ray source is irradiating the doors front on. Measurement error in each case is 0.01 μSv/h

### Absolute dosimetry

**Fig. 3:**
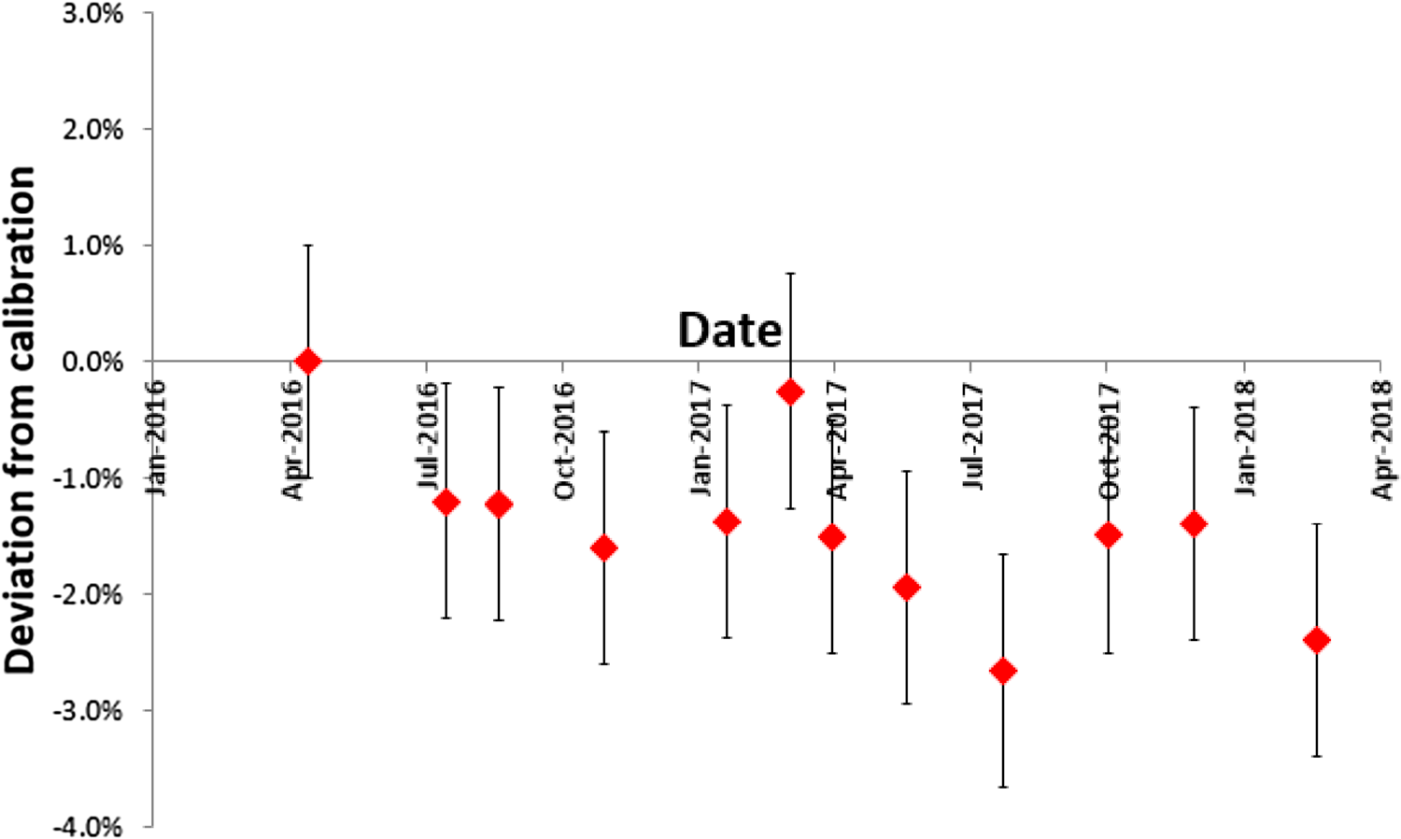
Output constancy with time at 225 kV, 13 mA. Error bars represent an estimated combined (baseline and constancy measurement) uncertainty of ± 1%

The 1st and 2nd HVL were found to be 1.05 mm Cu and 2.06 mm Cu respectively with an estimated 2% uncertainty (compared to values of 0.98, 0.91, 1.02, and 1.95, 1.89 and 2.14 respectively for the three centres reported by Lindsay et al.). The results for the absolute output are shown in Table 2. The results for the output constancy over time are presented in Fig. 3.

**Table 2:** Absolute output in air and in phantom for the 40 × 40 mm^2^ collimator at the system isocentre

	40 × 40 mm^2^ collimator	Lindsay et al.	Difference (%)
Output in air (Gy/min)	3.61 ± 0.05	3.67 ± 0.21	1.64
Output in phantom (Gy/min)	3.07 ± 0.05	3.17 ± 0.21	3.16
PDD at 2 cm depth (%)	75.00 ± 3.00	77.00 ± 2.00	2.60
TPR at 2 cm depth (%)	85.13 ± 3.00	87.67 ± 0.03	2.90
Phantom/air output (%)	85.14 ± 0.10	87.00 ± 0.01	2.14

The values from Lindsay et al. [8] are the means (± SD) from three institutions

**Fig. 4:**
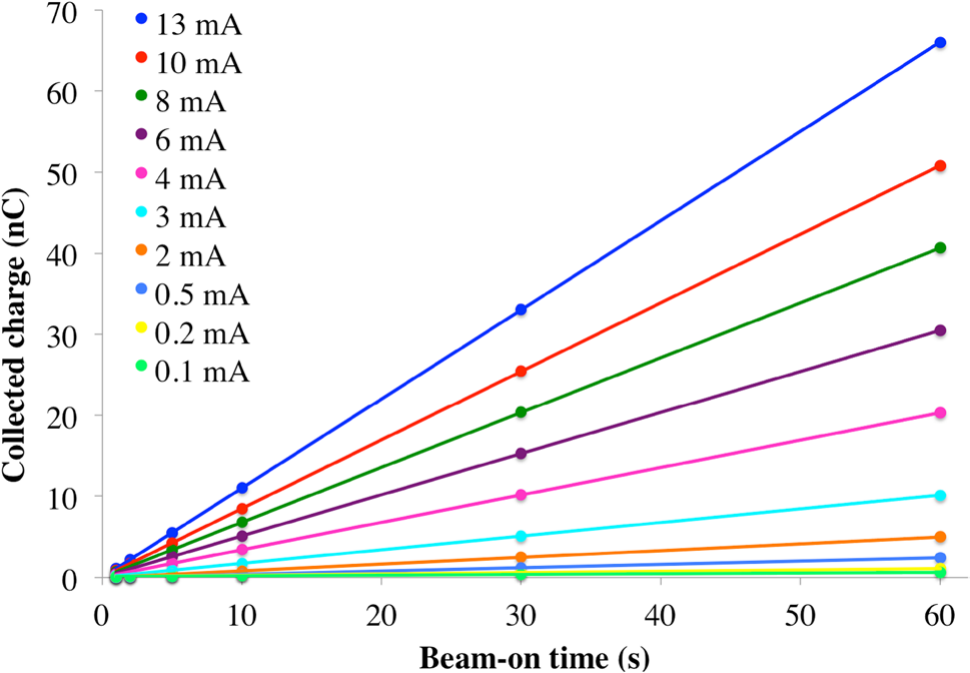
Linearity curve: charge versus beam-on time for multiple beam currents

The values obtained for dose linearity at different currents from 0.1 to 13 mA are presented in Fig. 4. From these the time error can be obtained as 0.116 s, which occurs at the smallest current of 0.1 mA with a linear fit of R^2^ = 0.981.

### Relative dosimetry

**Fig. 5:**
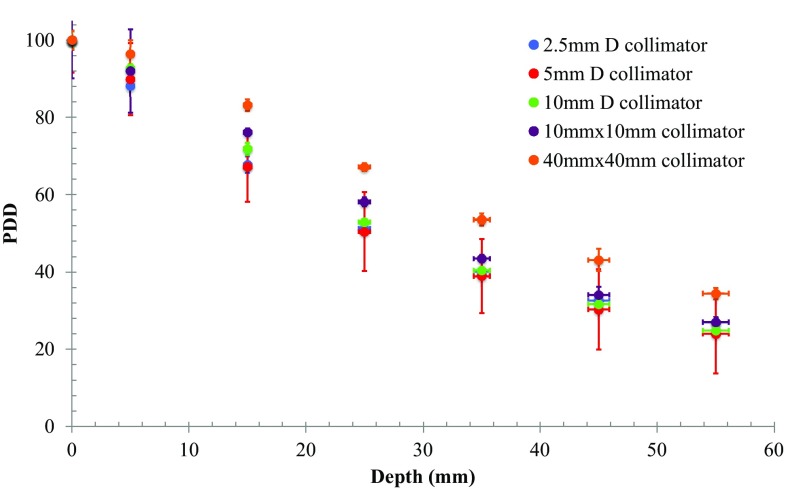
PDD data points for a range of field sizes, measured with EBT3 film

The uncertainty indicated in results is the standard deviation between the three films evaluated for each irradiation. Figure 5 shows the PDD curves for all collimators. PDD values from the SmART-Plan calculation (not presented) are within 3% PDD of measurement at all depths.

**Fig. 6:**
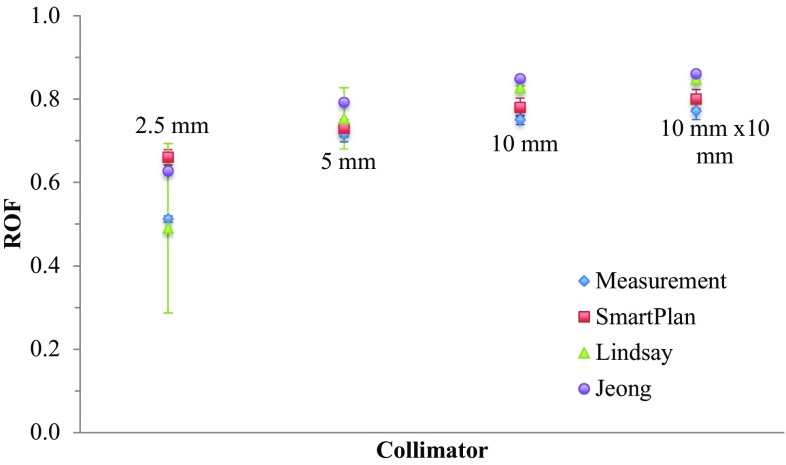
Relative output factors (measured, calculated and from the literature)

The results for the ROF measurements are presented in Fig. 6 for different field sizes relative to the 40 × 40 mm^2^ field, measured at 5 mm depth of solid water. Additionally, the values from Lindsay et al. [8] as well as Jeong et al. [11]. and those calculated in the planning system (SmartPlan) are presented for comparison.

A summary of all beam profile characteristics obtained from the profiles is presented in Table 3, compared with calculated profiles from SmART-Plan and those published.

**Table 3:** Relative dosimetry results x and y direction averaged, measured at 5 mm depth in solid water phantom

	Average	SD	Lindsay et al.	SD	Diff. (%)	TPS	Diff. (%)
2.5 mm							
Field size (mm)	2.64	0.06	2.53	0.28	4.1	2.25	14.8
Penumbra (mm)	1.06	0.02	0.53	0.19	49.8	0.64	39.8
Symmetry	6.23	0.79	–	–	–	–	–
Flatness	5.27	0.32	–	–	–	21.09	300.3
5 mm							
Field size (mm)	5.30	0.02	5.53	1.07	4.4	4.99	5.8
Penumbra (mm)	1.12	0.06	0.57	0.12	49.6	0.61	45.7
Symmetry	1.92	0.28	0.67	0.24	65.3	–	–
Flatness	5.41	0.21	4.83	3.32	10.7	22.79	320.9
10 mm							
Field size (mm)	10.34	0.00	10.00	0.34	3.3	10.23	1.1
Penumbra (mm)	1.01	0.05	0.57	0.15	44.0	0.60	40.7
Symmetry	1.05	0.45	2.03	1.48	94.3	–	–
Flatness	5.94	0.50	2.10	0.57	64.6	12.44	109.4
10 × 10 mm^2^							
Field size (mm)	10.44	0.02	10.27	0.12	1.7	10.36	0.8
Penumbra (mm)	1.08	0.06	0.63	0.08	41.5	0.61	43.6
Symmetry	1.76	0.59	1.07	1.01	39.2	–	–
Flatness	6.19	0.40	1.70	0.14	72.5	10.00	61.6
40 × 40 mm^2^							
Field size (mm)	39.53	0.38	41.50	2.63	5.0	42.08	6.4
Penumbra (mm)	1.44	0.15	1.43	0.90	0.2	0.84	41.5
Symmetry	0.21	0.01	3.93	2.14	1733.4	–	–
Flatness	5.46	0.31	4.73	0.68	13.3	14.66	168.5

The values from the film are presented against the values from Lindsay et al. [8], as well as calculated in the SmART-Plan TPS (interpolated from the dose grid). Differences in % are relative to the film measurements, being absolute change in symmetry and flatness. Symmetry values from the TPS only reflect calculation variance and are not indicated

**Fig. 7:**
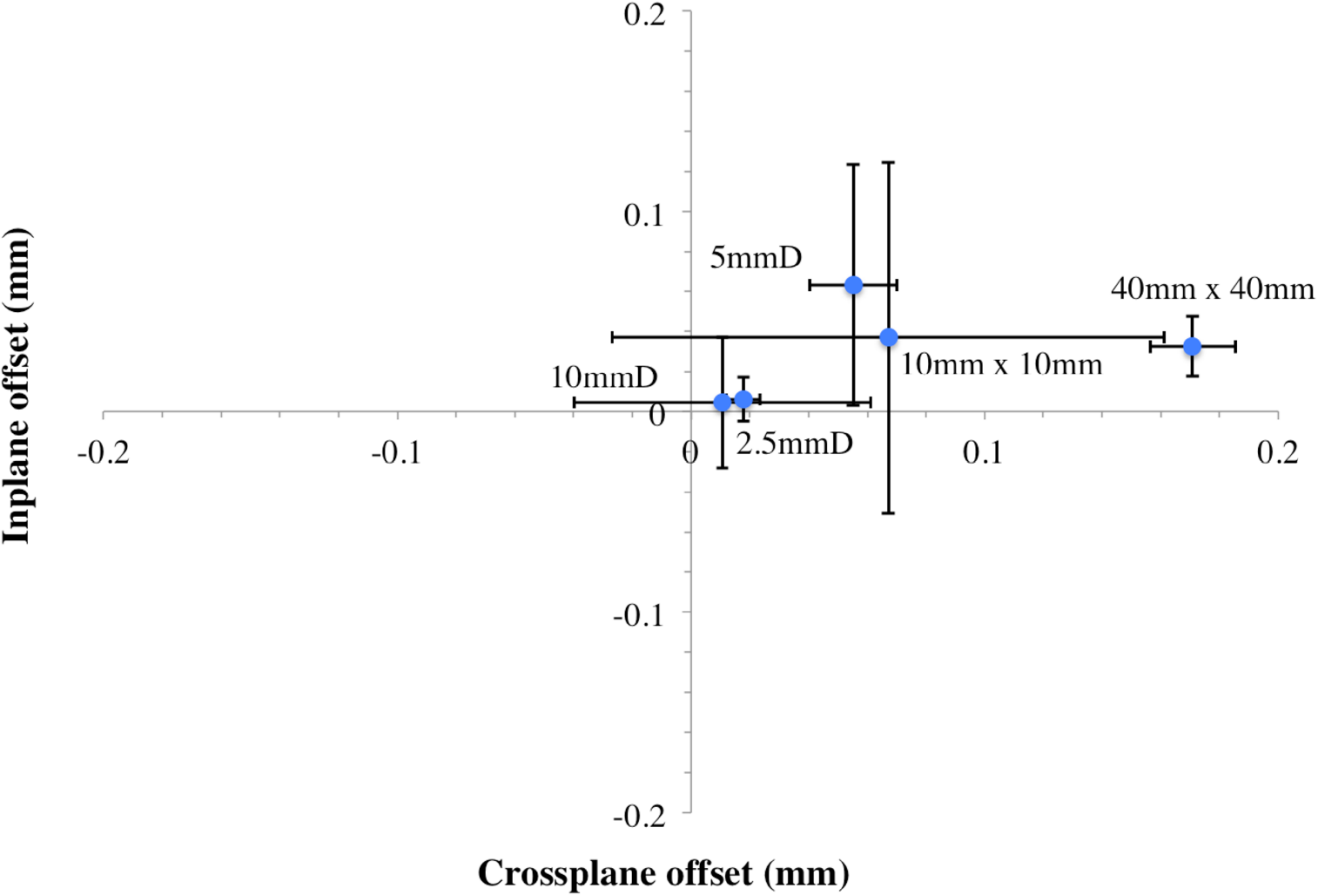
Effective offset of the source from isocentre in the x and y plane

The graph obtained for the effective offset of the X-ray source from isocentre in both planes is presented in Fig. 7.

For distances within 5 cm of the isocentre the effective SSD measured was 30.2 cm with R^2^ = 0.999.

### Mechanical operation and performance

**Fig. 8:**
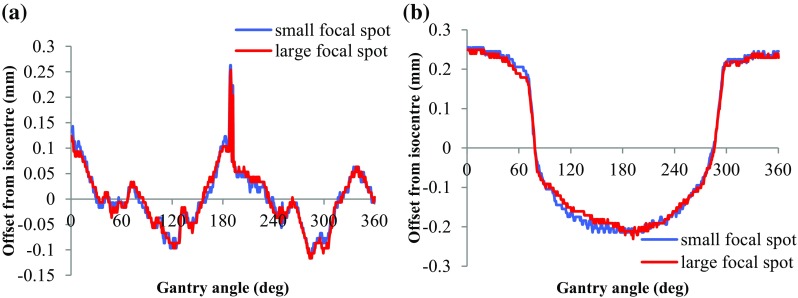
**a** Flex map in the u-axis (orthogonal to the axis of rotation). **b** Flex map in the v-axis (parallel to the axis of rotation of the system)

The magnification measurement indicated an SAD of 30.6 ± 0.1 cm, SDD to the imaging panel of 62.5 ± 0.1 cm resulting in a magnification factor of 2.04. The obtained flex maps are shown in Fig. 8 for the big (red line) and small (blue line) focal spot. The values are shown relative to the system isocentre.

**Fig. 9:**
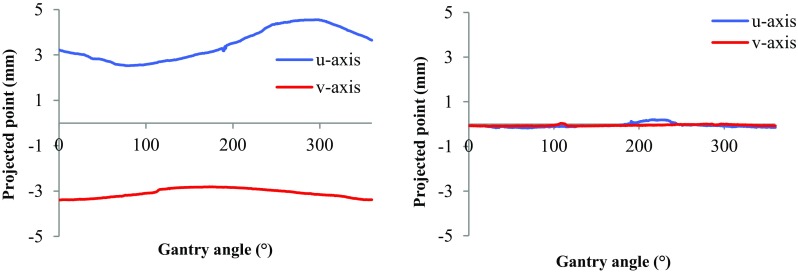
(Left) Projected point against gantry angle with stage tracking off. (Right) Projected point against gantry angle with stage tracking turned on

The results for the Winston-Lutz (W-L) test for *u* and *v* of the system with tracking off and on are shown in Fig. 9.

The magnitude of the flex in both *u* and *v* (with tracking off) has a maximum of 2.02 mm. The maximum residual motion (after W-L test with tracking on) in (*u, v*) is (0.2, 0.1) mm.

## Discussion

The results from this study indicate the performance characteristics of the X-RAD device installed at the TKI. Comparison with similar results published for devices from the same manufacturer provide indication of the variability likely in reports of experiments undertaken on such devices installed globally. The estimated uncertainties and variability in dose delivery has been poorly reported in small-animal studies [12]. The measurements performed and quantities presented here are intended to be comparable with the reporting requirements detailed in Desrosiers et al. [12].

### Absolute dosimetry

The absolute output dose-rate measured for the Telethon Kids Institute’s X-RAD device is consistent with those reported across other institutions by Lindsay et al. [8]. The beam quality, assessed with HVL, PDD and TPR, is also consistent across institutions, varying within acceptable uncertainties.

The output constancy presented in Fig. 3 showed some consistent decline over the 18 months of monitoring though has remained within the established tolerance, which is < 2% for two consecutive readings or < 3% for a single reading. Note that international standards for dosimetric traceability and uncertainty in pre-clinical IGRT systems have not yet been established and these tolerances have been arbitrarily selected. A recent survey of small-animal irradiators [13] indicated variations of 5% to 42% in expected dose delivery.

The time error of 0.116 s for the system investigated is consistent with those reported for the systems studied by Lindsay et al. [8] being less than 0.2 s and varying in sign and magnitude across the range of beam currents. Timer error was ignored in timer calculations.

### Relative dosimetry

The measured PDD data in Fig. 5 display expected characteristics for a 225 kVp beam, with scatter contributions increasing with increasing exposed beam area. Although not shown, measured depth-dose data was consistent with those calculated via the phantom simulation in SmART-Plan.

As can be seen in Fig. 6, there is considerable variation in output factor across the reported and measured values, with large variations in measured ROF for the smallest collimator. This effect also translates to a comparison of the measured and calculated ROFs for the Telethon Kids Institute’s X-RAD system, which, although within 3% for the other field sizes, is approaching a difference of 30% for the 2.5 mm circular collimator. This variation within a centre and between centres presents some considerable concern for dose delivery and reporting for experiments involving that smallest field size. Several factors could influence that consistency, including volume-averaging and uncertainties in the dose calculation (noting that in Fig. 7, error bars in the SmART-Plan values indicate standard deviation in the dose calculation), sensitivity to alignment of the X-ray focal spot and collimation system and focal spot shape, and discrepancies in collimator manufacture that are not accommodated by the planning system [8].

As presented in the quantified profile information in Table 3, field sizes differ from the average published values by 5% or less, however other measured dose profile quantities vary more widely. Penumbral widths differ considerably, though in absolute terms they vary between devices by less than 0.7 mm, and more consistently between the film measurements and SmART-Plan. It should be noted that the penumbral width measurement was sensitive to noise along the profile. The discrepancies could also be due to resolution difference; the use of 75 dpi scanning was a limitation for accurate penumbra assessment for such small fields, and higher resolution scanning is recommended in future studies. Differences between measured and published profile quantities become smaller for larger field sizes. For the 40 × 40 mm^2^ collimator, there is also considerable variability in penumbral measurements across the devices reported by Lindsay et al. Flatness was reduced (beam profiles were less flat) in the device relative to those reported for profiles by Lindsay et al., and SmART-Plan calculated values typically outside the uncertainty in measured values. Also, in comparing values in Table 3 it is assumed that interpretation of the derived quantities and their calculation are consistent with those by Lindsay et al.

Consistency in the field width and penumbral size was generally found between the x and y directions, though positions of the effective source offset for all collimators are grouped in the same quarter of the coordinate system as can be seen in Fig. 7. This is indicative of a systematic shift in the x-ray source from isocentre to the right in the in-plane and cross-plane directions.

The obtained value for the effective SSD at isocentre of 30.2 cm is slightly higher than the values found by Lindsay et al. The value is consistent with the measured SAD of 30.6 cm, given the presence of additional scatter sources.

### Mechanical operation and performance

The manufacturer specified an SAD of 30.64 cm, consistent with the value of 30.6 ± 0.1 cm for the Telethon Kids Institute’s X-RAD system. Lindsay et al. found values consistently between 30.1 cm and 30.2 cm for SAD and 62.0 cm and 62.3 cm and suggested that such consistency means standard values could be used across their systems. However, the sensitivity of image reconstruction to SAD and the 4 to 6 mm difference between their reported values and the Telethon Kids Insitute’s X-RAD system indicates that SAD and SSD need to be established at each institution independently.

The values obtained for gantry flex (Fig. 8) are consistent with those presented by Lindsay et al. Gantry flex measurements must be undertaken independently on each system as the image reconstruction algorithm uses the flex data to correct acquired planar images. In addition, the mechanical variations in isocentre with gantry rotation, quantified via Winston-Lutz test (Fig. 9) and showing oscillations of the order of 2 mm lateral to the beam axis, are automatically compensated for via movement (tracking) of the animal support stage. As such, with the tracking applied according to the isocentre pattern of Fig. 9a, the movement is reduced to < 0.2 mm as shown in Fig. 9b (within the resolution of the imaging system ~ 0.1 mm).

## Conclusion

This study enabled characterisation of a commercially available small-animal IGRT system and a comparison of its performance characteristics with similar devices. Basic dosimetric quantities are comparable between the system studied here and those reported elsewhere, with a notable exception being the output dose-rate for the smallest collimator (2.5 mm circular) where variation between systems from the same manufacturer have already been demonstrated. In addition, a large difference was found between expected and delivered dose for this collimator indicating that experiments utilising that collimator should consider the importance of accurate dose delivery and subsequent reporting of that delivery. By virtue of the automatic motion-compensation process incorporated into the X-RAD design, beam targeting accuracy throughout the gantry rotation can be achieved to within 0.2 mm. The device was considered acceptable for its intended purpose, though limitations of dosimetric and geometric accuracy are being considered during design of relevant experiments.

Although commissioning of the small-animal IGRT device mimicked aspects of commissioning of a comparable clinical IGRT system, physical restrictions on the design of the device, significantly different beam quality and dimensions, and higher spatial resolution mean some measurements and procedures needed to be customised. As these systems become more widely used, it is important that their dosimetric calibration and characterisation is consistently performed and reported so that subsequent small-animal experiments can be undertaken and stated with a similar level of consistency and comprehensiveness as clinical treatments. This study can hopefully form a part of the move to such consistency.
